# Leveraging National Cancer Institute Programmatic Collaboration for Uterine Cervix Cancer Brachytherapy in Puerto Rico After Hurricane Maria

**DOI:** 10.3389/fonc.2019.00414

**Published:** 2019-05-17

**Authors:** Charles A. Kunos, Percy Ivy

**Affiliations:** Cancer Therapy Evaluation Program, National Cancer Institute, Bethesda, MD, United States

**Keywords:** Puerto Rico, uterine cervix cancer, cervical cancer, brachytherapy, National Cancer Institute (NCI)

## Abstract

Evaluating the impact of natural disasters on cancer patients is vital to the recovery of cancer treatment services and infrastructure. In September 2017, Hurricane Maria demolished the gynecologic cancer service in the United States territory of Puerto Rico and its outreach to the territory of the Virgin Islands of the United States. Patient access to brachytherapy for uterine cervix cancer patients can be difficult to measure in the aftermath of a hurricane. The United States National Cancer Institute (NCI) surveyed gynecologic radiation medicine providers on the island to generate an independent perspective on gynecologic brachytherapy service recovery after the hurricane. Providers were asked about patient displacement, infrastructure loss, and reestablishment of cancer treatment. Here, the NCI provides its perspective on recovery of these services as it relates to its pre-hurricane investment for staff in the NCI Community Oncology Research Program.

## Introduction

The United States territory of Puerto Rico was devastated in September 2017 by the impact of two back-to-back hurricanes. While most of the island lost power after Hurricane Irma (1), it was the direct hit by the powerful Category 4 Hurricane Maria that caused catastrophic humanitarian damage (2). Maria's storm surge fouled lifelines such as water, food, shelter, roads; knocked down 80 percent of the island's electrical grid; destroyed 100 percent of cell phone service infrastructure; and interrupted timely commodity resupply (2). An estimated 4,150 or more residents in Puerto Rico were sheltered in congregate facilities for 30 days or more as they sought housing elsewhere on the island or on the United States mainland (3). Island-wide medical access and care capacity were reduced by 97 percent, including significant disruptions to the island's cancer services (3). Recovery of cancer treatment services and infrastructure in the aftermath of natural disasters remains of interest to the United States National Cancer Institute (NCI) for its public health service preparedness planning and future risk reduction to its clinical trial enterprise.

The NCI conducts trials in Puerto Rico supported by a Minority/Underserved NCI Community Oncology Research Program (PRNCORP) newly based at the Centro Comprensive de Cancer–Universidad de Puerto Rico (CCCUPR) Research Hospital, which opened in San Juan in June 2018 (4). The PRNCORP navigates women with advanced stage uterine cervix cancer undergoing radiochemotherapy through one of three San Juan-centric gynecologic brachytherapy centers, with only an estimated 30 percent of women undergoing the requisite brachytherapy (4). Pre-hurricane, the NCI provided resources to the PRNCORP to hire a gynecologic research nurse that would help steer uterine cervix cancer patients through treatment, inclusive of their brachytherapy, with a particular emphasis to meet requirements of NCI-sponsored uterine cervix cancer trials (like NRG Oncology GY006 that randomly allocates untreated women with advanced-stage uterine cervix cancer to triapine-cisplatin-radiotherapy vs. cisplatin-radiotherapy [NCT02595879]).

Here, the NCI provides an independent perspective on its post-hurricane plans for a PRNCORP gynecologic research nurse. To provide context, the NCI collected infrastructure loss and care delivery data in Puerto Rico from the initial 12-month period after Hurricane Maria. It also queried its PRNCORP providers about hurricane-related patient displacement, infrastructure loss, and reestablishment of gynecologic brachytherapy services (5–8).

## Challenges and Opportunities

Brachytherapy is a key element in the cure of advanced stage uterine cervix cancer and vitally improves survival (9, 10). The two most commonly practiced brachytherapy methods, high-dose-rate (HDR) and low-dose-rate (LDR) brachytherapy, are considered equivalent (9). NCI trials for advanced stage uterine cervix cancer like NRG Oncology GY006 allow either HDR or LDR brachytherapy. Pre-hurricane, HDR brachytherapy was practiced virtually in all cases in Puerto Rico or those patient referrals from the Virgin Islands of the United States (4), as the method affords outpatient treatment, avoids staff radiation exposure, allows reproducible applicator placement, and offers prescription dose optimization through adjustable source dwell-time steps. Inferior survival and increased toxicity rates are reported in women who receive higher doses of external-beam radiotherapy and lower or no brachytherapy doses (10–12).

### Barriers to Brachytherapy After Hurricane Maria

In the aftermath of Hurricane Maria, HDR brachytherapy services in Puerto Rico were suspended due to power outages and to patient transportation difficulties (7, 8). With loss of electricity, brachytherapy treatment issues immediately arise. Remote afterloaders for HDR brachytherapy require electricity to drive motors for stepping source cables that allow for the variable source dwell-times critical to the technique. Remote afterloaders themselves are mobile units but given the high radioactivity sources they use to delivery radiation dose, they require shielding to minimize staff or other individual radiation exposure during treatment relegating the machines to shielded vaults or dedicated rooms.

Medical images from computed tomography scanners, which draw upon electrical power, are used to judge adequacy of internal applicator placement, as brachytherapy treatment plan optimization cannot compensate for poor applicator positioning (9). Also, brachytherapy treatment plan optimization, or the sophisticated practice of attaining pre-specified radiation dose volumes to treat cancer and to minimize toxicity done at each individual treatment procedure (9), requires computers that also draw upon electrical power.

Some aspects of brachytherapy such as pretreatment patient or plan verification, record-and-verify dose delivery, and quality assurance management can be done without electrical power. But others like remote video camera medical monitoring of a patient during real-time HDR brachytherapy treatment need power. These issues were encountered on the island after Hurricane Maria knocked out the power grid (7, 8).

After natural disasters like hurricanes, the opportunistic use of LDR brachytherapy overcomes power-related issues in HDR brachytherapy. LDR brachytherapy applicators are manually after loaded with medical ^137^Cesium radioactive sources (10). From a radiobiology viewpoint, the low rate of radiation emission in LDR brachytherapy offers tumor cytotoxicity and normal tissue sublethal damage repair for reduced toxicity (10). The practical advantages of LDR brachytherapy are reduced reliance on electrical power to deliver brachytherapy, long-standing history of the technique with standardized (but still customizable) tandem and colpostat loading patterns, direct patient medical monitoring and care, and mobility as shields might just be rolling lead-based screens (10). Disadvantages might be provider unfamiliarity with the LDR technique, manual decay calculations to determine ^137^Cesium dwell time for a desired prescription dose, a requisite for three-dimensional over two-dimensional prescription planning, and protracted patient bed time (up to 40 h) to accommodate the low rate of radiation emission in LDR brachytherapy. LDR ^137^Cesium radioactive sources were not available for brachytherapy pre-hurricane or post-hurricane on the island (7, 8).

**Figure 1:**
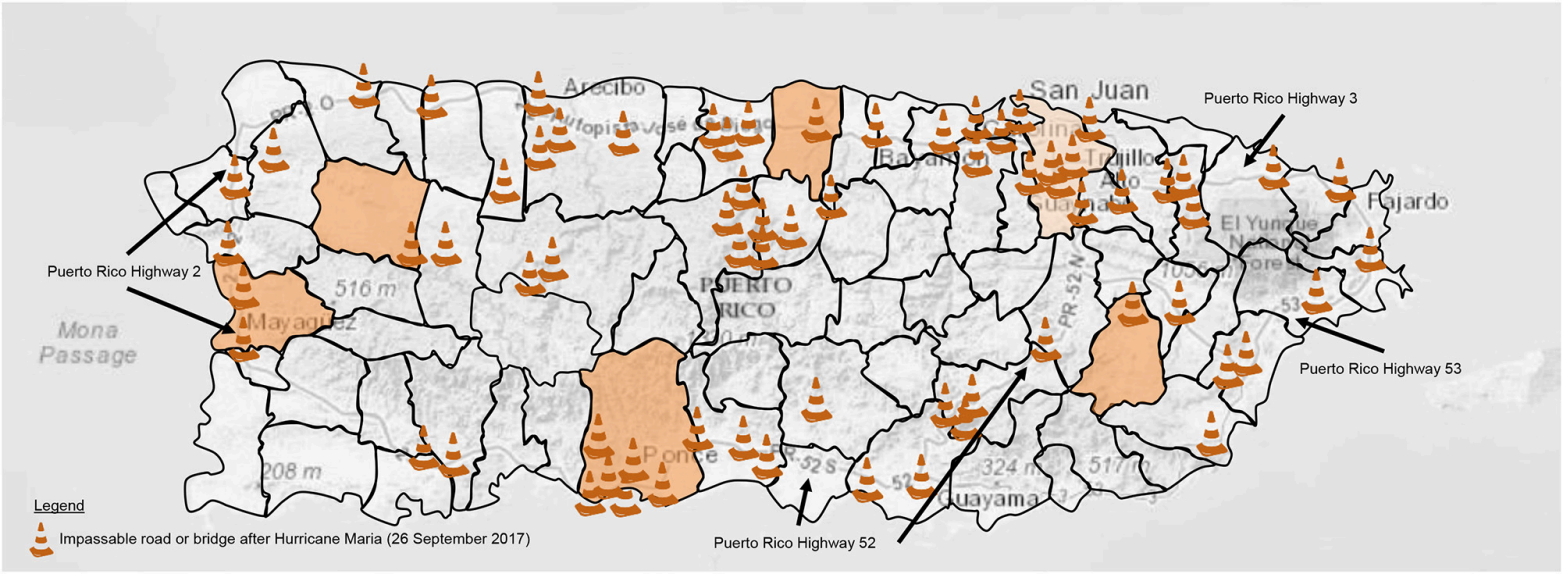
Depicted are the municipalities of the Commonwealth of Puerto Rico. Orange shading indicates age-adjusted rate of uterine cervix cancer per 100,000 population (2010–2015) in San Juan (12.6) or other high incident municipalities (>15.5) served by the PRNCORP on 26 September 2017 (http://www.rcpr.org/). Puerto Rico highways 2, 3, 52, and 53 are labeled as indicated. Impassable roads or bridges due to Hurricane Maria or Irma are indicated by traffic cone symbols as marked by the US Federal Emergency Management Agency and local crowdsourcing on 26 September 2017 (13, 14).

The second barrier to brachytherapy in the aftermath of Hurricane Maria was damage to roadway infrastructure (Figure 1). $70 million dollars and five bridges, requiring 800 metric tons of new steel, rebuilt roadways on the island swept away or deemed impassable after the hurricane (15, 16). Although it is well-established that urban women have greater odds of receiving brachytherapy than rural women (17), inequities in availability, accessibility, and utility reduce the likelihood of gynecologic brachytherapy for women with advanced stage uterine cervix cancer on the island. Over 80% of those women do not have access to a brachytherapy facility within 20 kilometers, and therefore, must rely on one of three San Juan-centric providers for brachytherapy (4). Lack of coordination, inadequate patient awareness for need of brachytherapy, and travel issues have contributed to a significant gap (70%) in the non-utilization of brachytherapy by women residing in rural municipalities in Puerto Rico (4). Evidence on the extent to which distance of travel acts as a deterrent to brachytherapy has not been well-studied. Lack of public transport, poor roads, and low perceived quality of care indicate a negative impact (18).

Before Hurricane Maria, women already sometimes spread out or skipped prescribed brachytherapy treatments altogether due to travel issues, according to some respondents (7, 8). One respondent said that these practices were already so commonplace that after the storm, given inadequate road infrastructure, women just did not return for brachytherapy despite pleas broadcast on public radio or television (7). Clearly, understanding barriers to brachytherapy use after a natural disaster is complex, but given its implicit associations with reduced survival with incomplete or no brachytherapy dose (10), damaged road infrastructure and disrupted telecommunications are factors that need consideration in a public health service preparedness plan on the island.

The NCI does not have an opportunity in a hurricane's aftermath to effect availability or accessibility of brachytherapy service. Consistent with patterns of brachytherapy practice on the American mainland (17), the density of brachytherapy service provision across a territory (in terms of travel time) impacts utilization. Before Hurricane Maria, the NCI identified a prospect to rectify the lack of brachytherapy service coordination on the island and outlined a plan for a dedicated research nurse coordinator to centralize access to brachytherapy (4). The ability of the PRNCORP and the CCCUPR to fill this dedicated research position has been stalled due to the practical limitations imposed by the Puerto Rico Oversight, Management, and Economic Stability Act (PROMESA, public law No: 114-187 [06/30/2016]) (6). Now after the storm, the NCI has the chance to work around this apparent roadblock through good clinical practice and patient education initiatives for the vital use and continuity of brachytherapy care.

Prior research indicates that messages directed toward patients that portray cancer treatment services, as part of trials or not, raise awareness that (a) state-of-the-art choices offer hope, (b) show women like themselves engage in such services for benefit, and (c) are explained in a clear, brief way with actionable steps for them to take (19). Targeted messages like these are needed in Spanish for women in Puerto Rico on active treatment for advanced stage uterine cervix cancer. The NCI has begun such an effort that is to be coordinated through the PRNCORP and CCCUPR.

## Perspectives on Gynecologic Brachytherapy After a Natual Disaster

Brachytherapy after a natural disaster poses a unique treatment challenge within a single treatment course, care for women with advanced stage uterine cervix cancer within a total of eight uninterrupted weeks (9). Indeed, survival decreases 0.6 percent for each additional day of treatment beyond 55 days for all stages of disease (20, 21). Typically, NCI's trials for uterine cervix cancer test new therapeutic agent questions, and thus, permit either integrated HDR or LDR brachytherapy for this reason. For Puerto Rico, respondents indicated that only HDR brachytherapy was available post-hurricane (7, 8). Power outages and roadway infrastructure losses were associated with delays in the recovery of brachytherapy services, suggesting that even a single storm may have a substantial impact upon patient treatment outcome.

The NCI plans further Hurricane Maria uterine cervix cancer treatment outcome research over the next several years. Factors that influence treatment outcomes considered for study include assessment of patient trust, family support, health literacy, access to transportation, availability of child care, and the logistics of alternate language consent form and translation services (22).

Several issues related to gynecologic brachytherapy should be considered here. First, this article considered uninterrupted brachytherapy, in a Spanish-language uterine cervix cancer population, on the territorial islands of Puerto Rico and the Virgin Islands of the United States together. This is because women with uterine cervix cancer from the Virgin Islands of the United States seek cancer care in San Juan-based cancer clinics or hospitals (7). Ongoing studies are evaluating the feasibility of cancer patient evacuation prior to or in the aftermath of natural disasters like hurricanes. Second, the NCI assessed suitability of its recommended brachytherapy prescription for women with bulky uterine cervix cancer, as this is the most common disease presentation in Puerto Rico. The conventional 6 Gy for five gynecologic brachytherapy fractions, with allowable variation (9), remains steadfast. Third, a small number of lifelines greatly impact brachytherapy service preparedness in Puerto Rico. Disruption of five lifelines—water, shelter, power, telecommunications, transportation—were found to meaningfully prohibit recovery of brachytherapy use on the island. The practicality of LDR brachytherapy in Puerto Rico is being reported elsewhere (7). How these items impact other cancer care services is being comprehensively studied in other disease and clinical trial contexts.

While recovery of lifeline resources in the aftermath of natural disasters is beyond the scope of the NCI, the NCI can strategize with its stakeholders to develop generalizable cancer patient preparedness plans. For instance, the NCI, with CCCUPR and PRNCORP staff, might enable means to coordinate near-term stays in its affiliated research hospitals or a nearby American Cancer Society Hope Lodge. NCI also might collaborate with other Federal agencies for plans to ensure access to diesel fuel electricity generators or means to sterilize brachytherapy applicators. In addition, the NCI might furnish Spanish-language patient information that stresses the importance of uninterrupted cancer care, or, support a gynecologic research nurse as part of a team to reestablish accessible brachytherapy centers until post-hurricane lifelines recover.

## Conclusion

This article offers unique insights on how technical limitations and travel influences brachytherapy care delivery among women with advanced stage uterine cervix cancer in Puerto Rico in the aftermath of a hurricane. Respondents to NCI inquiries indicated that power outages blocked the immediate recovery and damaged roads delayed long-term reestablishment of brachytherapy services on the island. Post-hurricane, one of the strategies to restore brachytherapy service is to provide patient education resources that describe clearly the importance of brachytherapy as it relates to their survival from uterine cervix cancer. Staff charged with coordinating cancer treatments island-wide (like a dedicated gynecologic research nurse navigator) positively impacts the likelihood of service recovery and emphasizes a need for active engagement to ensure brachytherapy service quality in Puerto Rico.

Further studies should be carried out in Puerto Rico to assess whether similar patterns are apparent for other cancer types. The same analysis could apply to other types of services for which travel time is a critical factor (e.g., urgent airway management for head and neck cancer cases) to provide evidence of the impact of natural disasters on healthcare utilization across territories like Puerto Rico and the Virgin Islands of the United States.

## Data Availability

No datasets were generated or analyzed for this study.

## Ethics Statement

The research presented in this article involved the collection or study of existing data, documents, and records that were publicly available, or the information was recorded by NCI in such a manner that trial subjects cannot be identified directly or through identifiers linked to the subjects. The research is regarded exempt from Institutional Review Board oversight.

## Author Contributions

CK and PI contributed to the collection and review of any data, analysis, and authentication, and the writing and approval of this manuscript. The views expressed are those of the authors and not those of the U.S. federal government. Links or discussions of specific treatments do not constitute endorsement.

### Conflict of Interest Statement

The authors declare that the research was conducted in the absence of any commercial or financial relationships that could be construed as a potential conflict of interest.
